# Developing the Spanish Version of the Fraboni Scale of Ageism: Cross-Cultural Adaptation with Initial Reliability and Content Validity Findings

**DOI:** 10.3390/clinpract16060104

**Published:** 2026-05-31

**Authors:** Juan Ramón de-Moya-Romero, Alexis Caballero-Bonafé, Laura Fernández-Puerta, Raquel Valera-Lloris, Antonio Martínez-Sabater

**Affiliations:** 1 Nursing Care and Education Research Group (GRIECE), Nursing Department, Faculty of Nursing and Podiatry, University of Valencia, 46010 Valencia, Spain; demoya_jua@gva.es (J.R.d.-M.-R.); caballero_alebon@gva.es (A.C.-B.); antonio.martinez-sabater@uv.es (A.M.-S.); 2 Care Research Group (INCLIVA), Valencia Clinic Hospital, 46010 Valencia, Spain; 3 Doctoral Program in Nursing Science, Jaume I University, 12006 Castelló, Spain

**Keywords:** ageism, instrument validation, psychometrics, healthcare professionals

## Abstract

**Background:** Ageism is a global public health concern associated with poorer health outcomes and inequities in care. Culturally adapted instruments are needed to assess ageist attitudes among healthcare professionals in Spain. This study aimed to cross-culturally adapt and evaluate the preliminary psychometric properties of the Spanish version of the Fraboni Scale of Ageism (FSA-SV). **Methods:** A methodological study was conducted, including translation and back-translation, expert review, and a pilot test. Content validity was assessed using the content validity index (CVI), the modified kappa coefficient, and Aiken’s V. A descriptive cross-sectional pilot study was conducted with 101 healthcare professionals from a single health department in Valencia to evaluate comprehension and reliability. Internal consistency was examined using Cronbach’s alpha and McDonald’s omega. **Results:** Content validity indices indicated acceptable agreement among experts (S-CVI = 0.745; Aiken’s V = 0.770). All items were retained to preserve conceptual and structural equivalence with the original instrument. The FSA-SV demonstrated high internal consistency (Cronbach’s alpha = 0.903; McDonald’s omega = 0.915). The mean total score was 51.2 (SD = 9.62), with no significant associations observed between ageism and participants’ sociodemographic or professional variables. **Conclusions:** This pilot study represents a first step in the cross-cultural adaptation and preliminary psychometric evaluation of the FSA-SV for use among healthcare professionals in Spain. The results suggest that the instrument shows promising initial properties for the preliminary assessment of ageism, supporting its potential utility in future research and in evaluating educational and organizational interventions aimed at reducing ageism and improving the quality and safety of care for older adults. Further studies with larger, more diverse samples are required to evaluate additional psychometric properties, including the factorial structure.

## 1. Introduction

Population ageing is one of the most significant demographic changes of the twenty-first century, driven by continued improvements in life expectancy and declining fertility rates. This situation is leading to an unprecedented increase in the proportion of older adults worldwide. The World Health Organization (WHO) estimates that the number of persons aged 60 years or older will double between 2020 and 2050, and the number of persons aged 80 years or older will triple [1]. These changes pose major challenges for health and long-term care systems [2]. This trend is associated with increasing dependency ratios and growing demand for long-term care, due to the higher risk of frailty, vulnerability, and multimorbidity in old age [3,4,5].

Within this demographic context, ageism has emerged as a priority public health concern. The World Health Organization (WHO) defines ageism as age-based discrimination encompassing stereotypes, prejudice, and discriminatory practices directed at individuals based on age. Globally, approximately one in two people holds ageist attitudes, with direct consequences for the health, quality of life, and rights of older adults [4].

Empirical evidence has consistently linked ageism to poorer physical and mental health outcomes, increased social isolation, reduced well-being, a higher risk of premature mortality, and substantially greater healthcare costs [6,7,8,9]. Systematic reviews further support these associations across their various manifestations and multiple health indicators, including impaired psychological and physical functioning and engagement in health-risk behaviours.

In addition, internalized negative attitudes toward ageing may adversely affect treatment adherence, physical activity, and social participation in later life [10,11].

Ageism also manifests in healthcare settings, where differential treatment, symptom underestimation, reduced access to specific therapies, and the underrepresentation of older adults in clinical trials contribute to health inequities [12]. These practices not only affect clinical outcomes but are also associated with lower quality of care, poorer clinician–patient communication, suboptimal decision-making, and an increased risk of adverse events among older adults, thereby undermining patient safety and care equity [7].

In Spain, one of the most aged countries worldwide, projections indicate that by 2068, older adults will account for approximately 29.4% of the population, further amplifying the structural impact of these dynamics [13]. During the COVID-19 pandemic, media narratives also reinforced stereotyped and paternalistic portrayals of old age [14], contributing to the social normalization of ageism.

Recognizing the scale of the problem, the United Nations General Assembly and the World Health Organization (WHO) declared the Decade of Healthy Ageing (2021–2030), identifying the elimination of ageism as a strategic priority. In line with this, a systematic review by Ayalon et al. [15] highlights the need for valid instruments to measure the phenomenon, monitor its evolution, and evaluate interventions aimed at reducing it.

Psychometrically sound and culturally adapted instruments are needed for rigorous assessment. While there are some established measures, such as Kogan’s scale and the Facts on Aging Quiz, many of these assess only partial dimensions of attitudes or focus primarily on cognitive components [16]. The Fraboni Scale of Ageism (FSA) is characterized by its multidimensional approach, combining cognitive, affective, and behavioral components, and by its predictive capacity for discriminatory behaviors [17].

The scale has been translated and validated across multiple languages and cultural contexts, demonstrating adequate psychometric properties [18,19,20,21,22,23,24]. It comprises 29 items rated on a four-point Likert scale (1 = strongly disagree; 4 = strongly agree), yielding total scores ranging from 29 to 116, with higher scores indicating more negative attitudes toward older adults. It should be noted that items 16, 19, 20, 21, 22, 25, and 27 are reverse-worded and were therefore reverse-coded before the calculation of total scores to ensure consistent interpretation.

The FSA operationalizes the affective component of ageism based on three of Allport’s five levels of prejudice: antilocution (antipathetic talk), avoidance (tendency to avoid older adults), and discrimination (exclusion from rights or opportunities), represented by 10, 10, and 9 items, respectively.

However, despite growing evidence of ageism’s impact on health and care quality, there is currently no validated, multidimensional instrument for the Spanish context that enables measurement comparable to that in other countries. This gap hampers the systematic identification of the problem, the evaluation of training interventions, and the development of evidence-informed policy. A culturally adapted Spanish version is therefore essential to generate comparable evidence, identify ageist attitudes among healthcare professionals, and evaluate interventions designed to improve the quality and safety of care for older adults. Cross-cultural adaptation helps ensure that observed differences between populations reflect true variation in the construct rather than linguistic or cultural artifacts. Accordingly, this study aimed to translate and cross-culturally adapt the FSA into Spanish, and to examine its content validity and preliminary reliability, in a sample of healthcare professionals.

## 2. Materials and Methods

### 2.1. Design

This study employed a methodological design to evaluate the content validity and reliability of the Spanish version of the FSA, following international recommendations for the cross-cultural adaptation of psychological instruments [25]. Comparable methodologies have been used for instrument adaptation, often with study-specific modifications, by multiple groups [26,27,28,29,30]. Permission to adapt the original scale was obtained from the developers via e-mail. We then implemented a multi-phase protocol to produce the Spanish version of the FSA.

### 2.2. Translation and Back-Translation

The original English FSA [17] was translated into Spanish by two independent bilingual healthcare professionals with advanced English proficiency. Working separately, each produced a draft, which a gerontology expert subsequently reconciled to yield a single, consensus Spanish version (FSA-SV). During reconciliation, semantic, idiomatic, conceptual, and experiential equivalence were assessed.

The consensus FSA-SV was then back-translated into English by two university nurse professors with expertise in questionnaire validation, generating two independent back-translations. A third bilingual translator compared each back-translation with the original FSA on an item-by-item basis to detect discrepancies. Questions regarding the consensus wording and the interpretability of items raised during the back-translation review were resolved via e-mail with the original authors, resulting in the final consensus FSA-SV.

### 2.3. Experts Review

The expert panel was selected through convenience sampling, based on predetermined criteria, including professionals with at least 5 years of experience in aging, gerontology, or geriatrics, and representing both clinical and academic settings. We also explicitly considered each candidate’s track record in teaching, research, or interventions targeting attitudes towards aging, to ensure that appraisals remained focused on the construct of ageism rather than geriatric practice alone. Experts rated the suitability and relevance of each FSA-SV item on a 5-point scale (1 = not suitable/not relevant; 5 = very suitable/very relevant).

### 2.4. Content Validity Analysis

Following established methodological recommendations for content validity assessment (e.g., Polit and Beck) [27,29,30,31,32] the content validity of the questionnaire was analyzed by calculating the content validity index (CVI), modified kappa coefficient (k) and Aiken’s V value for each item and the global questionnaire, which was based on the ratings made by the group of experts, using the following equations [33]:

(a) Content validity index (CVI)
  CVI = number of experts who evaluated the item with 4 or 5 (A)/total number of experts (N)
(b) Modified kappa (*k*)
  $$k=\frac{\left(I-CV\;I\right)-Pc}{1-Pc}$$

The I-CVI is the coefficient of internal validity, previously calculated for each item. In contrast, the Pc (probability of chance agreement) is the probability of chance agreement between observers and is calculated through the following formula:

$$Pc\;=\left[\frac{\left[N!\right]}{\left[A!\left(N\;-\;A\right)!\right]}\right]\;x0.5^{N}$$

(c) Aiken’s V. Its equation, algebraically modified by Penfield and Giacobbi (2004), is as follows [34]:
  $$V=\frac{X-l}{k}$$

X is the mean of the experts’ ratings, l is the lowest possible score, and k is the range of possible values of the Likert scale used. For example, if the lowest score is 1 and the highest score is 5, then k = 5 − 1 = 4. Once calculated, Aiken’s V confidence intervals were obtained using the scoring method [35], with the following formula being used to calculate the lower limit of the interval:

$$L=\frac{2nkV\;+\;z^{2}\;-\;z\;\sqrt{4nkV(1\;-\;V)\;+\;z^{2}}}{2(nk\;+\;z^{2})}$$

and for the upper limit of the interval:

$$U\;=\frac{2nkV\;+\;z^{2}+\;z\;\sqrt{4nkV(1\;-\;V)\;+\;z^{2}}}{2(nk\;+\;z^{2})}$$

L: lower limit of the interval; U: upper limit of the interval; z: value in the standard normal distribution; V: Aiken’s V calculated by formula c; n: number of experts.

The CVI, modified kappa, and Aiken’s V were calculated using a database created in Excel 365, based on the expert group’s assessments and their respective formulas.

### 2.5. Internal Consistency Analysis

The preliminary investigation (pilot test) allowed an assessment of the questionnaire’s internal consistency using Cronbach’s alpha and McDonald’s omega. A threshold of 0.7 of Cronbach’s alpha and McDonald’s omega is considered the minimum acceptable value, with scores below this indicating insufficient internal consistency of the instrument. For the calculation of Cronbach’s alpha and McDonald’s omega, the JAMOVI 2.6.44 statistical software was employed.

### 2.6. Pilot Study

A descriptive cross-sectional study, conducted among healthcare professionals in Spain, was designed to use the FSA-SV for the first time and to identify comprehension issues. Item comprehensibility was systematically assessed using a five-point Likert scale ranging from 1 (“Totally incomprehensible”) to 5 (“Totally comprehensible”). To evaluate item-level and overall questionnaire comprehensibility, mean scores were calculated for each item and for the total scale. This quantitative approach allowed us to identify potential comprehension issues at both the item and questionnaire levels during the pilot phase. Comprehensibility levels above 3.5 were considered adequate. Participants in this pilot study were also asked to reflect on any misunderstandings or difficulties encountered with the FSA-SV.

Participants of this pilot study were also asked to comment on any misunderstandings or difficulties experienced with the FSA-SV. Healthcare professionals actively working during the study period in specialized or primary care within one health department of the Valencian health system in Spain were expected to be included in the pilot study. The purpose was to collect at least 100 questionnaires through convenience sampling. The normality assumption for continuous variables was assessed using the Shapiro–Wilk test in the bivariate analysis. Continuous outcomes were compared between groups using Student’s *t*-test or the Mann–Whitney U test, as appropriate for the data distribution. Additionally, to explore the association between quantitative variables and the total FSA-SV score and its dimensions, Pearson or Spearman correlation coefficients were calculated based on the variables’ normality. For these analyses, JAMOVI 2.6.44 statistical software was employed.

### 2.7. Data Collection

#### 2.7.1. Expert Evaluation

Experts were contacted via e-mail, provided with information about the study, and invited to participate. Upon agreement, the consensus version of the FSA-SV was sent to them, along with instructions to evaluate the adequacy and comprehensibility of each item. They were also invited to suggest alternative wording where appropriate.

Participation was voluntary, anonymous, and confidential, and data were collected using a questionnaire administered via the Microsoft Forms platform. Experts who agreed to participate provided informed consent in accordance with current Spanish data protection regulations. Data collection took place between September and October 2025.

#### 2.7.2. Pilot Study

The pilot study was conducted through an ad hoc questionnaire distributed via e-mail to health professionals working in a Health Department of the Valencian health system. The participants were selected through convenience sampling. Before completing the questionnaire, they were asked to provide informed consent, which explained the purpose of the pilot study and the voluntary nature of their participation. All responses were collected anonymously, and no personally identifiable information was requested or stored, which complies with the European Union’s General Data Protection Regulation and the Spanish Organic Law 3/2018 on the Protection of Personal Data and Guarantee of Digital Rights. Microsoft Forms ensured secure data transmission and storage via encrypted channels and restricted access to the research team via institutional credentials.

In the first section, sociodemographic and professional data were collected (age, sex, academic level, profession, years of professional experience, specific training in the last 5 years). In the second part of the questionnaire, the FSA-SV was attached. In addition to responding to the questionnaire, they could also identify any problems with the items’ comprehension and provide their personal comments.

The pilot study data were collected from October to December 2025. This group’s participation was voluntary, anonymous, and confidential, and was conducted via a questionnaire on the Microsoft Forms platform.

### 2.8. Ethical Considerations

The study was approved by the Research Ethics Committee of the University Clinical Hospital of Valencia (reference 2024/344). Participation was voluntary and anonymous, and confidentiality of the data was fully ensured. Access to the data was restricted exclusively to the research team.

## 3. Results

### 3.1. Results of the Translation Process

During translation from English to Spanish, some scale items required clarification. Regarding item seven, the term “be irritating” did not translate smoothly into “ser irritantes,” so the item was reworded as “ser cargantes,” capturing the sense of discomfort that may arise from the constant repetition of the same ideas. As for item 21, “Old people should be encouraged to speak out politically”, the initial translation rendered it as “Se debería animar a las personas mayores a hablar más en política”; however, given that the item is intended to capture the expression of personal viewpoints rather than political engagement in a broader sense, the wording was ultimately revised to “Se debería animar a las personas mayores a manifestar sus opiniones sobre política”. The results of the translation of the FSA-SV are shown in Table 1.

### 3.2. Characteristics of Experts

Fifteen experts responded to the questionnaire. Of these, 53.3% (8) were women. The mean age of the participants was 47.1 years with a standard deviation (SD) of 8.26. The mean years of professional experience was 23.9 years, with a SD of 7.64; the experience in geriatric care was 16.7 years, with an SD of 7.44. Regarding their academic degree, 66.7% (*n* = 10) held a PhD, 6.7% (*n* = 1) had a master’s degree, and 26.7% (*n* = 4) were graduates. Overall, 80% (*n* = 12) of the experts had specific training in geriatric care and gerontology. Regarding the participants’ working environment, most respondents reported performing their duties in non-geriatric clinical units (33.3%; *n* = 5). A smaller proportion indicated working specifically within a geriatric care unit (13.3%; *n* = 2). An additional 13.3% (*n* = 2) of participants occupied non-clinical roles, such as intermediate management or administrative positions. Finally, the largest group, 40% (n = 6), selected “academic/university” as their area of activity.

### 3.3. Content Validity Analysis

The content validity assessment of the FSA-SV demonstrated overall acceptable agreement among experts across the evaluated indices. The scale-level Content Validity Index (S-CVI) was 0.745, with a modified Kappa coefficient of 0.726 and an overall Aiken’s V of 0.770, indicating an acceptable-to-good content validity. At the item level, several elements exhibited suboptimal performance relative to commonly recommended criteria for adequate content validity (i.e., I-CVI ≥ 0.78 and Aiken’s V ≥ 0.70–0.75). Specifically, items 1, 7, 8, 15, 16, and 17 yielded the lowest indices, with I-CVI values ranging from 0.600 to 0.667, Kappa coefficients from 0.528 to 0.633, and Aiken’s V values between 0.683 and 0.750. Despite these lower coefficients, no items were removed.

The adaptation process aimed to preserve the conceptual content and original item composition of the Fraboni Scale, thereby maintaining theoretical continuity and facilitating future cross-cultural comparisons. Consequently, items with lower performance were subjected to qualitative refinement (linguistic and semantic review) rather than exclusion, ensuring conceptual comparability with the source instrument during this preliminary adaptation phase.

Comprehensibility levels above 3.5 were considered adequate; therefore, no items were removed for this reason.

Table 2 shows CVI, Aiken’s V, and comprehensibility values for each questionnaire item.

### 3.4. Pilot Study Participants

The study sample consisted of *n* = 101 healthcare professionals. The mean age of participants was 47.4 years (SD = 10.60), ranging from 24 to 69 years. The total years of professional experience averaged 21.4 years (SD = 9.96; range: 1–40), and years of specific experience in geriatrics averaged 4.8 years (SD = 7.93; range: 0–37). The average time spent in the current job position was 7.7 years (SD = 7.33; range: 0–34).

Regarding categorical variables, the sample was predominantly female (89.1%; 90). Additionally, 80.2% (*n* = 81) of the professionals reported not having completed specific training on ageing in the past five years, compared with 19.8% (*n* = 20) who indicated having participated in such training. Regarding the professional profile, most participants worked as nurses (72.3%; *n* = 73). Other roles represented in the sample included nursing assistants 12.9% (*n* = 13), midwives 4.95% (*n* = 5), physicians 4.95% (*n* = 5), physiotherapists 2.0% (*n* = 2), occupational therapists (1.0%) (*n* = 1), laboratory technicians (1.0%) (*n* = 1), and supervisory staff (1.0%) (*n* = 1).

### 3.5. Reliability Analysis

Following the pilot test, internal consistency was assessed using Cronbach’s alpha and McDonald’s omega based on responses from participating healthcare professionals. The FSA-SV demonstrated excellent internal consistency (Cronbach’s alpha = 0.903; McDonald’s omega = 0.915). Neither coefficient increased substantially following the removal of any individual item, as shown in Table 3.

The FSA-SV exhibited adequate internal consistency across its dimensions. The Antilocution dimension demonstrated good reliability, with Cronbach’s alpha of 0.831 and McDonald’s omega of 0.836. Similarly, the Discrimination dimension demonstrated satisfactory internal consistency, with Cronbach’s alpha of 0.728 and McDonald’s omega of 0.793. The Avoidance dimension also showed good reliability, with a Cronbach’s alpha of 0.734 and a McDonald’s omega of 0.772, further supporting the scale’s internal coherence. Table 4 shows the reliability scores for each dimension when a dimension is deleted.

### 3.6. Pilot Study Results

Regarding the FSA-SV results, the participants’ average score was 51.2. Table 4 shows the results in each dimension of FSA-SV.

The FSA-SV scores showed very similar values across sexes. Among men, the mean of total FSA-SV’s score was 50.0 (SD = 6), while the dimension avoidance score was 17.1 (SD = 3.70), discrimination was 14.2 (SD = 2.48), and antilocution was 18.8 (SD = 3.52). Among women, the corresponding scores were 51.3 (SD = 9.97) for the total FSA-SV score, 16.2 (SD = 3.65) for avoidance, 14.9 (SD = 3.13) for discrimination, and 20.2 (SD = 4.49) for antilocution. These differences were not statistically significant, as indicated by Mann–Whitney U tests for total score (U = 467, *p* = 0.485), discrimination (U = 456, *p* = 0.410), avoidance (U = 462, *p* = 0.448), and antilocution (U = 379, *p* = 0.102). Similarly, the scores according to specific training in aging were practically equivalent. The group without training scored 51.0 (SD = 9.62) on FSA-SV’s total score, 16.3 (SD = 3.60) on avoidance, 14.9 (SD = 3.08) on discrimination, and 19.8 (SD = 4.45) on antilocution, whereas those who had received such training obtained 51.8 (SD = 9.90), 16.4 (SD = 3.95), 14.5 (SD = 3.02), and 20.8 (SD = 4.23), respectively. No statistically significant differences were observed here either, as reflected in the Mann–Whitney U test values for total score (U = 780, *p* = 0.798), discrimination (U = 765, *p* = 0.700), avoidance (U = 798, *p* = 0.918), and antilocution (U = 670, *p* = 0.231).

Age did not show significant Spearman correlations with FSA-SV’s total score (Rho = 0.123, *p* = 0.220), Avoidance (Rho = 0.159, *p* = 0.112), Discrimination (Rho = 0.187, *p* = 0.062), or Antilocution (Rho = 0.007, *p* = 0.946). Likewise, years of professional experience were unrelated to total (Rho = 0.110, *p* = 0.275), Avoidance (Rho = 0.139, *p* = 0.166), Discrimination (Rho = 0.144, *p* = 0.151), and Antilocution (Rho = 0.023, *p* = 0.822). The same pattern was observed for years of specific experience in geriatrics, which showed no significant associations with total (Rho = −0.121, *p* = 0.227), Avoidance (Rho = 0.132, *p* = 0.190), Discrimination (Rho = −0.102, *p* = 0.308), and Antilocution (Rho = −0.082, *p* = 0.413).

## 4. Discussion

Ageism in healthcare settings has been linked with differential treatment and barriers to accessing health services [12]. It has also been linked to worse physical and mental health outcomes in older adults [7,10] and increased healthcare costs [6]. In the context of accelerated population aging and growing evidence of ageism’s impact on health and care quality, the availability of valid, culturally appropriate measurement instruments is essential [6,7,10]. The FSA is a widely used instrument applied across multiple countries and diverse populations [17,20,21,22,23,24,36,37,38]. However, the questionnaire has not yet undergone cross-cultural adaptation and psychometric evaluation in Spain, despite its widespread use. Therefore, this study aimed to perform cross-cultural adaptation and to evaluate the preliminary psychometric properties of the FSA in a pilot sample of health professionals in Spain.

Establishing a valid Spanish version of the FSA will facilitate the evaluation of training interventions and policy initiatives aimed at reducing ageism in clinical settings. To our knowledge, this is the first cross-cultural adaptation and preliminary psychometric assessment of the FSA-SV in the Spanish healthcare context.

Regarding the content validity of the FSA-SV, 17 items showed values below optimal thresholds (I-CVI < 0.78), although all achieved at least moderate expert agreement (I-CVI ≥ 0.60). These findings indicate that content validity at the item level remains limited and should be interpreted with caution. In cross-cultural adaptation studies, items with lower indices are frequently retained and subjected to qualitative revision to preserve conceptual equivalence with the original instrument during preliminary adaptation phases [32,33,39]. Accordingly, items with lower performance were refined linguistically and semantically to improve clarity and cultural appropriateness without altering their conceptual meaning [25,31,40].

Some of the items with lower content validity indices may also reflect sociocultural particularities in perceptions of aging in the Spanish context. Specifically, items related to social avoidance, coexistence, or social exclusion of older adults from mainstream social environments may be interpreted differently in Mediterranean cultures, where intergenerational contact and family coexistence continue to play an important social role, unlike in more individualistic societies. Previous literature has suggested that ageism is closely associated with processes of social exclusion, reduced social participation, and stereotyped representations of dependence in later life, all of which are strongly shaped by cultural and community norms [40,41]. In this sense, some items related to avoidance or separation from older adults may not reflect only explicit ageist attitudes but also culturally mediated perceptions of caregiving roles, intergenerational relationships, and social participation in older age. Moreover, ageism has been linked to poorer health outcomes, reduced quality of life, and barriers to social inclusion among older adults across different healthcare and community settings [7]. These findings support the need for further qualitative and psychometric studies to explore how age-related stereotypes and social exclusion are conceptualized and expressed across different sociocultural contexts and healthcare environments.

At the scale level, the overall content validity index (S-CVI) was slightly below the commonly recommended 0.80 threshold (0.745). In our study, the S-CVI value (0.745) was lower than that reported by Fan et al. [20], who obtained an S-CVI of 0.93 with six experts, and lower than the 0.98 reported by Kutlu et al. [21] based on a panel of 12 nursing academics.

However, as noted by Polit and Beck [42], CVI cut-off points should not be applied rigidly, and values close to the recommended threshold may be acceptable when maintaining equivalence with the original version is a priority and when the expert panel size is moderate, given its influence on index stability. Furthermore, the mean kappa coefficient and Aiken’s V exceeded acceptable benchmarks for content validity [32,42]. Importantly, the present study, a pilot aimed at evaluating content validity, does not address other essential measurement properties, such as structural validity, test–retest reliability, convergent and discriminant validity, or measurement invariance across groups. Consequently, retaining all items was consistent with the need to preserve the original theoretical model and to enable future evaluation of factorial invariance and measurement equivalence across cultural contexts, an essential condition for international comparisons [43]. Early item removal based solely on expert judgment might improve content validity indices in the short term. However, it could compromise the instrument’s structural stability and limit empirical testing of the model through confirmatory factor analysis in larger samples [44].

At this preliminary stage, the goal is to preserve the conceptual content and item composition of the original FSA while identifying potential semantic or cultural comprehension issues that require refinement before definitive psychometric validation. In this context, exploratory or confirmatory factor analyses were intentionally deferred, since modifications derived from pilot testing could still affect the final structure of the Spanish version. Future studies with larger and more representative samples will be necessary to examine whether the factorial structure reported for the original FSA is replicated in the Spanish context.

To assess internal consistency, both Cronbach’s alpha and McDonald’s omega were calculated. The original FSA demonstrated adequate internal consistency (Cronbach’s alpha = 0.86) [17], whereas the present study yielded high values (Cronbach’s alpha = 0.903). The FSA-SV also showed higher internal consistency than the Chinese, Canadian, Turkish, and U.S. versions [17,20,21,22,24,36,37,38], surpassing only the Czech validation (alpha = 0.949) [23]. These findings suggest that, despite cultural differences, the items retain adequate internal coherence. Specifically, the Antilocution dimension (α = 0.831) in the present study exhibited markedly strong reliability, exceeding the values reported in the original validation by Fraboni et al. [17] (α = 0.76), the U.S. adaptation by Rupp et al. [37] (α = 0.79 for stereotypes), and the value documented by Li et al. [24] among Chinese long-term caregivers. Similarly, the Discrimination (α = 0.728) and Avoidance (α = 0.734) dimensions demonstrated solid reliability, consistently exceeding early benchmarks of 0.65 and 0.77 established by Fraboni et al. [17] and the 0.70 and 0.76 reported by Rupp et al. [37].

Comparative analysis of the FSA-SV’s reliability omega further underscores its robustness relative to the comprehensive psychometric benchmarks established by Remr [23]. The total scale achieved a McDonald’s omega of 0.915, closely approximating the 0.949 reported in the Czech general population. At the subscale level, omega coefficients remained strong—antilocution (ω = 0.836), discrimination (ω = 0.793), and avoidance (ω = 0.772). Although these values are slightly lower than the respective 0.92, 0.88, and 0.88 reported by Remr, such differences are methodologically predictable. The larger, more heterogeneous sample in the Czech study (n = 1096) likely contributed to higher reliability estimates. In contrast, the greater homogeneity of the present sample of healthcare professionals typically yields more conservative yet still excellent reliability values without compromising the instrument’s suitability for clinical research.

In the pilot study conducted among Spanish healthcare professionals, the mean total FSA-SV score was 51.2 (SD = 9.62), with subscale means of 31.3 for avoidance, 14.8 for discrimination, and 31.6 for antilocution, suggesting comparatively lower levels of ageist attitudes within this sample.

Compared with previous studies using the FSA, the present results suggest lower levels of ageism among nursing students than those reported by Rababa et al. [45], with a mean score of 69.5.

Similarly, studies conducted in the general population have consistently reported higher FSA scores, indicating greater ageism. For example, Remr [23] reported a mean total score of 65.65 (SD = 15.05) in a Czech population, whereas Kutlu [21] reported a mean total score of 59.66 (SD = 9.40) in a Turkish sample.

The absence of significant correlations between ageism and participants’ age, years of professional experience, or experience in geriatrics is consistent with findings from recent studies involving healthcare professionals [46,47,48]. One possible explanation is that ageism may depend less on the quantity of chronological or professional experience and more on the quality of intergenerational contact and the acquisition of specific gerontological knowledge, which may act as more robust protective factors against age-related stereotypes [49,50].

Additionally, the relative homogeneity of the pilot sample, predominantly female (89.1%) and largely composed of nurses (72.3%), may have reduced the variability required to detect statistically significant associations. These results suggest that other individual factors, such as anxiety about one’s own ageing or intrinsic motivation to work with older adults, may have greater explanatory value than professional experience alone.

In conclusion, the main finding of this study is that the Spanish version of the FSA-SV demonstrates high internal consistency and preliminary evidence of content validity among healthcare professionals, supporting its potential usefulness as an instrument to assess ageism in this context. From an applied perspective, having a Spanish version of the FSA with adequate preliminary psychometric properties has important implications for healthcare training and management. The systematic measurement of ageism enables the identification of attitudinal dimensions that can be targeted in interventions and the assessment of educational programs aimed at enhancing the quality of care for older people, which is particularly important in light of evidence associating ageist attitudes with worse health outcomes and poorer clinical relationships [7,9,10].

This study, therefore, represents a first step toward the systematic assessment of ageism among healthcare professionals in Spain, using a multidimensional instrument with preliminary psychometric support. Its availability enables international comparisons and provides a valuable tool for designing and evaluating educational and organizational interventions to reduce ageism and enhance the quality of care for older adults. While the availability of a Spanish-adapted version of the FSA represents a valuable contribution, further research is needed to comprehensively evaluate its psychometric properties and confirm its suitability for use in both research and clinical settings. In this sense, the present study should be understood as a foundational phase that supports, but does not complete, the validation process of the instrument.

### 4.1. Study Limitations

Regarding the sample of healthcare professionals, it is worth noting that the sample size used aligns with established recommendations for pilot studies. However, the composition of the sample, predominantly female, mostly nurses, and recruited from a single geographic region, limits the representativeness of the findings. Consequently, further studies involving larger, more diverse samples are needed to more robustly assess the reliability of the FSA-SV and to enhance the generalizability of the results to the broader population of healthcare professionals in Spain.

Additionally, the use of self-reported measures may introduce social desirability bias, as participants may tend to provide responses that are perceived as socially acceptable rather than reflecting their true attitudes. This issue may be particularly relevant in the context of ageism, given the increasing awareness of age-related discrimination and the normative expectation of respectful attitudes toward older adults within healthcare settings. Consequently, the observed levels of ageism may be underestimated. Future studies could address this limitation by incorporating complementary assessment methods, such as implicit measures or mixed-method approaches, to obtain a more comprehensive understanding of ageist attitudes.

### 4.2. Implications for Practice

The availability of the FSA-SV enables its integration into both undergraduate and continuing education for healthcare professionals, facilitating the detection and monitoring of ageist attitudes, contributing not only to raising awareness among practitioners but also to developing more effective training strategies.

Moreover, the independent assessment of the cognitive, affective, and avoidance components of ageism provides valuable data for developing targeted educational interventions to change specific elements of attitudes toward older adults. From an institutional perspective, the quantitative data generated by the FSA-SV offer healthcare and educational administrators compelling evidence to guide decisions and cultivate more respectful and inclusive clinical and educational environments. Such contributions can generally improve the human and technical quality of care delivered by healthcare systems.

## 5. Conclusions

The FSA-SV shows preliminary adequate internal consistency and acceptable content validity, representing an important first step towards the systematic assessment of ageism among healthcare professionals in Spain. Furthermore, larger, more diverse samples are needed in future multicenter studies to confirm the scale’s dimensional structure and establish its applicability in both research and clinical practice. Nevertheless, the existence of this instrument is an important step forward, as it provides the means to assess educational interventions and policy measures to combat ageism in healthcare contexts. Ongoing psychometric refinement of the instrument will be critical to enhancing its utility in research and clinical practice and to advancing evidence-based strategies to address ageism.

## Figures and Tables

**Table 1:** Spanish translation of FSA.

Item 1	SPA: Muchas personas mayores son tacañas y acumulan su dinero y posesiones ENG: Many old people are stingy and hoard their money and possessions
Item 2	SPA: Muchas personas mayores no están interesadas en hacer nuevos amigos, ya que prefieren conservar el círculo de amigos que han tenido siempre ENG: Many old people are not interested in making new friends, preferring instead the circle of friends they have had for years
Item 3	SPA: Muchas personas mayores viven permanentemente en el pasado ENG: Many old people just live in the past
Item 4	SPA: No se debería confiar en la mayoría de las personas mayores para cuidar de los bebés ENG: Most old people should not be trusted to take care of infants
Item 5	SPA: Muchas personas mayores son más felices cuando están con personas de su misma edad ENG: Many old people are happiest when they are with people their own age
Item 6	SPA: La mayoría de las personas mayores tienen mala higiene corporal ENG: Most old people would be considered to have poor personal hygiene
Item 7	SPA: Muchas personas mayores pueden ser irritantes, ya que cuentan las mismas historias una y otra vez ENG: Most old people can be irritating because they tell the same stories over and over again
Item 8	SPA: Las personas mayores se quejan más que otras más jóvenes ENG: Old people complain more than other people do
Item 9	SPA: Si me invitaran, preferiría no ir a una jornada de puertas abiertas en un club de mayores ENG: I would prefer not to go to an open house at a senior’s club‚ if invited
Item 10	SPA: El suicidio de adolescentes es más trágico que el suicidio de personas mayores ENG: Teenage suicide is more tragic than suicide among the old
Item 11	SPA: A veces evito establecer contacto visual con personas mayores cuando las veo ENG: I sometimes avoid eye contact with old people when I see them
Item 12	SPA: No me gusta cuando las personas mayores intentan entablar una conversación conmigo ENG: I don’t like it when old people try to make conversation with me
Item 13	SPA: No se puede esperar una conversación compleja e interesante de la mayoría de las personas mayores ENG: Complex and interesting conversation cannot be expected from most old people
Item 14	SPA: Es un sentimiento común sentirse deprimido cuando se está cerca de personas mayores ENG: Feeling depressed when around old people is probably a common feeling.
Item 15	SPA: Las personas mayores deberían encontrar amigos de su misma edad ENG: Old people should find friends their own age
Item 16	SPA: Las personas mayores deberían sentirse bienvenidas en las reuniones sociales de los jóvenes ENG: Old people should feel welcome at the social gatherings of young people
Item 17	SPA: Las personas mayores no necesitan usar las instalaciones deportivas comunitarias ENG: Old people don’t really need to use our community sports facilities
Item 18	SPA: Es mejor que las personas mayores vivan donde no molesten a nadie ENG: It is best that old people live where they won’t bother anyone
Item 19	SPA: La compañía de la mayoría de las personas mayores es muy agradable ENG: The company of most old people is quite enjoyable.
Item 20	SPA: Es triste escuchar hoy en día la situación de las personas mayores en nuestra sociedad ENG: It is sad to hear about the plight of the old in our society these days
Item 21	SPA: Se debería animar a las personas mayores a manifestar sus opiniones sobre política ENG: Old people should be encouraged to speak out politically
Item 22	SPA: La mayoría de las personas mayores son interesantes y únicos ENG: Most old people are interesting‚ individualistic people
Item 23	SPA: Personalmente, no querría pasar mucho tiempo con una persona mayor ENG: I personally would not want to spend much time with an old person
Item 24	SPA: Deberían existir instalaciones deportivas específicas para personas mayores, para que estas puedan competir con otras personas de su mismo nivel ENG: There should be special clubs set aside within sports facilities so that old people can compete at their own level
Item 25	SPA: Las personas mayores merecen los mismos derechos y libertades que los demás miembros de nuestra sociedad ENG: Old people deserve the same rights and freedoms as do other members of our society
Item 26	SPA: No se debería permitir a la mayoría de las personas mayores renovar sus permisos de conducir ENG: Most old people should not be allowed to renew their driver’s licenses
Item 27	SPA: Las personas mayores pueden ser muy creativas ENG: Old people can be very creative
Item 28	SPA: Preferiría no vivir con una persona mayor ENG: I would prefer not to live with an old person
Item 29	SPA: Las personas mayores no necesitan mucho dinero para cubrir sus necesidades ENG: Old people do not need much money to meet their needs

**Table 2:** CVI, Aiken’s V, and comprehension values.

ITEM No.	CVI	K	Aiken’s V	Aiken’s V CI 95%	Comprehensibility
1	0.667	0.633	0.683	(0.558–0.787)	3.733
2	0.733	0.722	0.783	(0.664–0.869)	4.267
3	0.800	0.797	0.767	(0.646–0.856)	4.267
4	0.733	0.722	0.733	(0.610–0.829)	4.133
5	0.800	0.797	0.783	(0.664–0.869)	4.267
6	0.800	0.797	0.767	(0.646–0.856)	4.267
7	0.667	0.633	0.750	(0.628–0.842)	4.267
8	0.600	0.528	0.717	(0.592–0.815)	4.133
9	0.800	0.797	0.733	(0.610–0.829)	4.200
10	0.800	0.797	0.833	(0.720–0.907)	4.400
11	0.733	0.722	0.750	(0.628–0.842)	4.200
12	0.800	0.797	0.767	(0.646–0.856)	4.333
13	0.733	0.722	0.750	(0.628–0.842)	4.200
14	0.733	0.722	0.750	(0.628–0.842)	4.200
15	0.600	0.528	0.733	(0.610–0.829)	4.267
16	0.667	0.633	0.717	(0.592–0.815)	4.000
17	0.600	0.528	0.733	(0.541–0.773)	3.867
18	0.800	0.797	0.767	(0.646–0.856)	4.333
19	0.867	0.866	0.850	(0.739–0.919)	4.400
20	0.733	0.722	0.800	(0.628–0.882)	4.133
21	0.667	0.633	0.767	(0.646–0.856)	4.133
22	0.867	0.866	0.817	(0.701–0.894)	4.133
23	0.733	0.722	0.783	(0.664–0.869)	4.333
24	0.667	0.633	0.733	(0.610–0.829)	4.000
25	0.800	0.797	0.817	(0.701–0.894)	4.267
26	0.667	0.633	0.750	(0.628–0.842)	4.133
27	1.000	1.000	0.950	(0.863–0.983)	4.733
28	0.800	0.797	0.850	(0.739–0.919)	4.533
29	0.733	0.722	0.767	(0.646–0.856)	4.067
TOTAL	0.745	0.726	0.770	(0.651–0.857)	4.214

CI: Confidence Interval; CVI: Content Validity Index; K: Kappa index.

**Table 3:** Reliability characteristics of FSA-SV.

Item No.	Correlation Coefficient: Item-Total Score	Cronbach’s Alpha If the Item Is Deleted	McDonald’s Omega If the Item Is Deleted
1	0.556	0.898	0.911
2	0.446	0.901	0.913
3	0.514	0.899	0.912
4	0.524	0.899	0.912
5	0.373	0.902	0.914
6	0.499	0.900	0.913
7	0.519	0.899	0.912
8	0.615	0.897	0.910
9	0.473	0.900	0.912
10	0.481	0.900	0.913
11	0.600	0.898	0.910
12	0.528	0.900	0.911
13	0.590	0.899	0.910
14	0.641	0.898	0.909
15	0.460	0.900	0.913
16 ^a^	0.331	0.903	0.914
17	0.568	0.899	0.910
18	0.552	0.900	0.910
19 ^a^	0.528	0.899	0.911
20 ^a^	0.374	0.902	0.914
21 ^a^	0.528	0.899	0.911
22 ^a^	0.344	0.902	0.914
23	0.572	0.899	0.910
24	0.234	0.907	0.916
25 ^a^	0.551	0.899	0.910
26	0.349	0.903	0.915
27 ^a^	0.466	0.900	0.912
28	0.532	0.899	0.912
29	0.414	0.901	0.913

^a^ reverse-code item.

**Table 4:** Scores of FSA-SV.

n = 101	Mean	Median	SD	Min	Max	Q1	Q3
Total	51.2	52	9.63	29	68	44	59
Avoidance	16.3	16	3.65	10	23	14	20
Discrimination	14.9	15	3.06	9	25	13	17
Antilocution	20	20	4.4	10	31	17	23

Min = minimum; Max = maximum; Q1 = first quartile; Q3 = third quartile; SD = standard deviation.

## Data Availability

The data presented in this study are available on request from the corresponding authors. The data are not publicly available due to privacy or ethical restrictions.

## Abbreviations

CI	Confidence Interval
CVI	Content Validity Index
ENG	English
FSA	Fraboni Scale of Ageism
FSA-SV	Spanish version of the Fraboni Scale of Ageism
I-CVI	Item-level Content Validity Index
*k*	Kappa index
MAX	Maximum
MIN	Minimum
Q1	First quartile
Q3	Third quartile
S-CVI	Scale-level Content Validity Index
SD	Standard deviation
